# Supplementary material for: Epigenetic-based differentiation therapy for Acute Myeloid Leukemia
Source: Nat Commun. 2024 Jul 2;15:5570. doi: 10.1038/s41467-024-49784-y (PMC11219871; doi:10.1038/s41467-024-49784-y)
Supplement: Supplementary file 1 — Supplementary Information [file 41467_2024_49784_MOESM1_ESM.pdf]

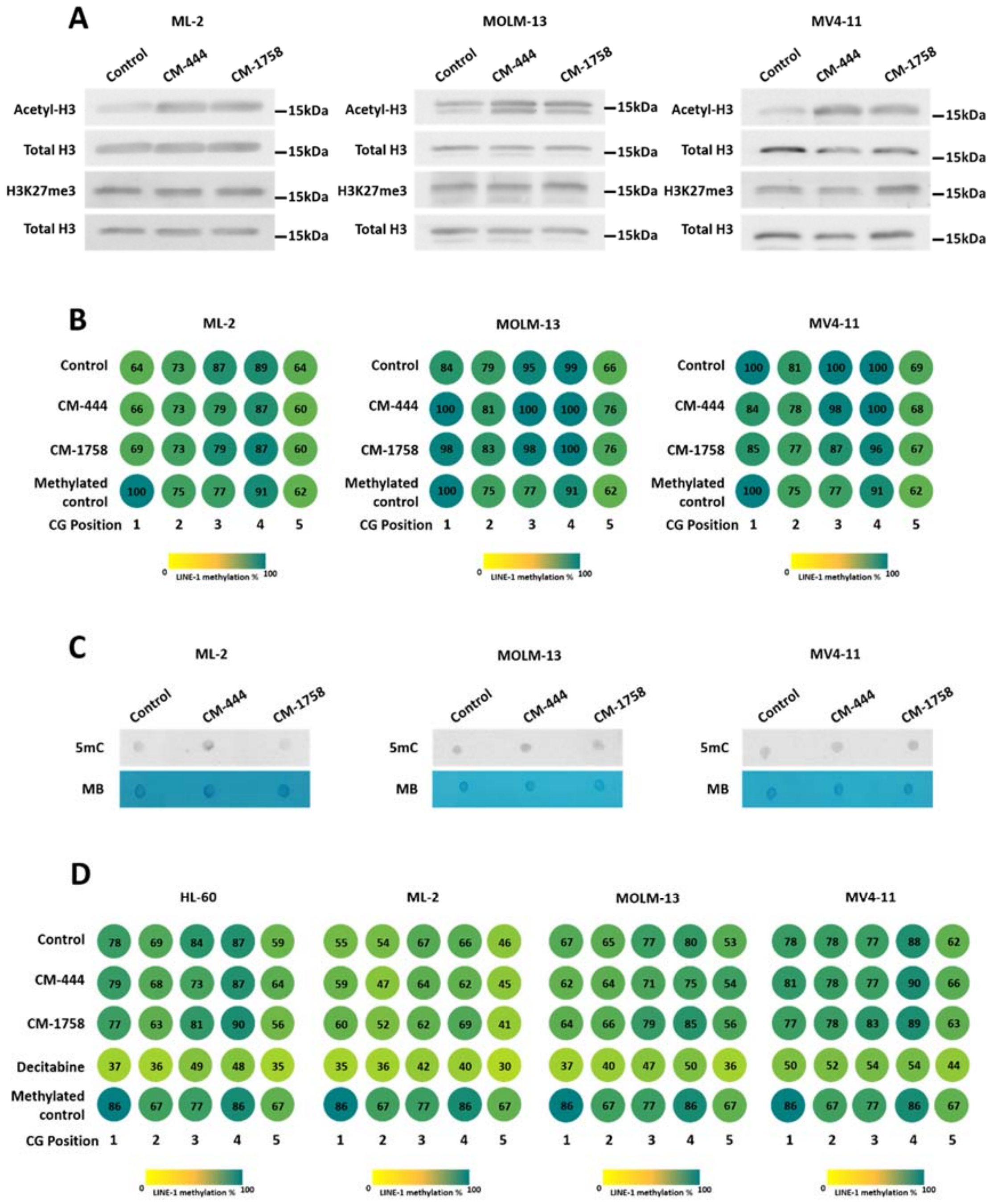

Supplementary Figure 1: Treatment of AML cells with CM-444 and CM-1758, selective pan-HDACi at low non-cytotoxic doses. ML-2, MV4-11, and MOLM-13 cell lines were treated daily for 48 hours with 260, 160, and 280 nM of CM-444 and 210, 80, and 140 nM of CM-1758, respectively. **A)** H3aC and H3K27me3 levels were detected by western blot after treatment. The experiment was repeated twice with similar results. **B)** DNA methylation analysis by pyrosequencing of *LINE*-1 was performed after treatment. Universally methylated DNA was used as DNA methylated control. The DNA methylation percentage is indicated inside the circles. The data shown the mean of two biologically independent experiments (n=2). **C)** Dot blot was used to detect 5-methylcytosine levels after treatment. Methylene blue staining was used as a loading control. The experiment was repeated twice with similar results. 5 mC: 5-methylcytosine; MB: methylene blue. **D)** DNA methylation of *LINE-1* analyzed by pyrosequencing after daily treatment with CM-

444 or CM-1758 in HL-60 and ML-2 cell lines for 10 days or MOLM-13 and MV4-11 cells for 5 days. The DNA methylation percentage is indicated inside the circles. Treatment with decitabine was used as a positive control of DNA demethylation. As a DNA methylated control, a universally methylated DNA was used. The data shown are the mean of two biologically independent experiments (n=2). Uncropped blots and source data are provided as a Source data file.

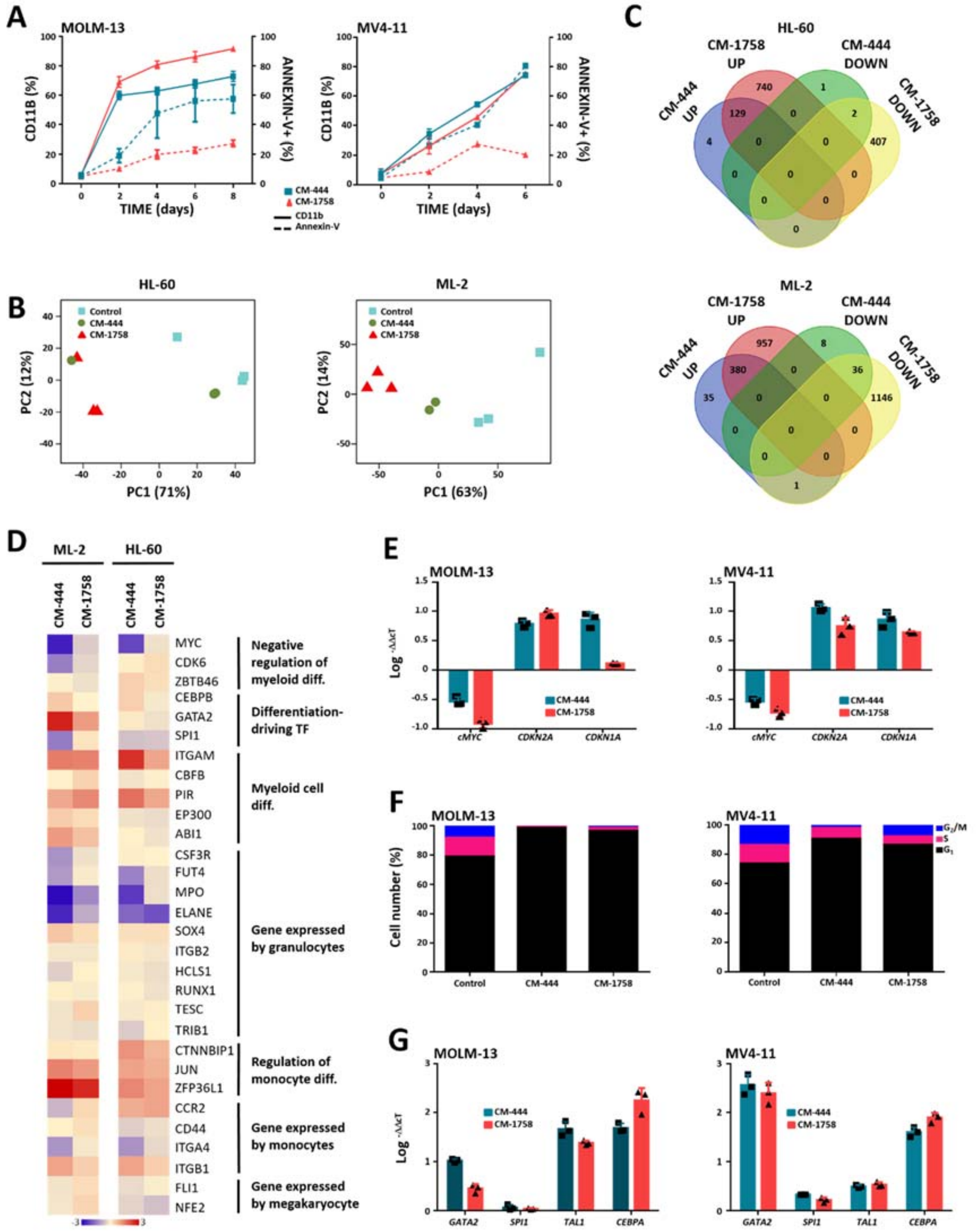

Supplementary Figure 2: Induction of cell differentiation in all subtypes of AML cells by CM-444 and CM-1758. A) CD11b and annexin-V were measured by flow cytometry at 2, 4, 6, and 8 days after daily treatment of MOLM-13 and MV4-11 cell lines with CM-444 (280 and 160 nM, respectively) and CM-1758 (140 and 80 nM, respectively). Data are presented as mean values +/- S.D. of three biological replicates. **B)** PCA of RNA-seq data from HL-60 and ML-2 cells after treatment with CM-444 or CM-1758 compared with untreated cells. **C)** Venn Diagram of differentially expressed genes after CM-444 or CM-1758 treatment in HL-60 and ML-2 cell lines. **D)** ML-2 and HL-60 RNA-Seq heat map showing the genes differentially expressed after CM-444 and CM-1758 treatments and related to myeloid differentiation and expression patterns of granulocytes and monocytes. **E–G)** q-PCR of *MYC*, *CDKN2A* (*p16*), and *CDKN1A* (*p21*);

(**E**), cell-cycle analysis; (**F**) and q-PCR of *GATA2*, SPI1 (*PU.1)*, TAL1 (*SCL),* and *CEBPA;* (**G**) in MOLM-13 and MV4-11 cell lines after treatment with CM-444 (280 and 160 nM, respectively) or CM-1758 (140 and 80 nM, respectively) for 48 hours. Data are presented as mean values +/- S.D. of three biological replicates. Source data are provided as a Source data file.

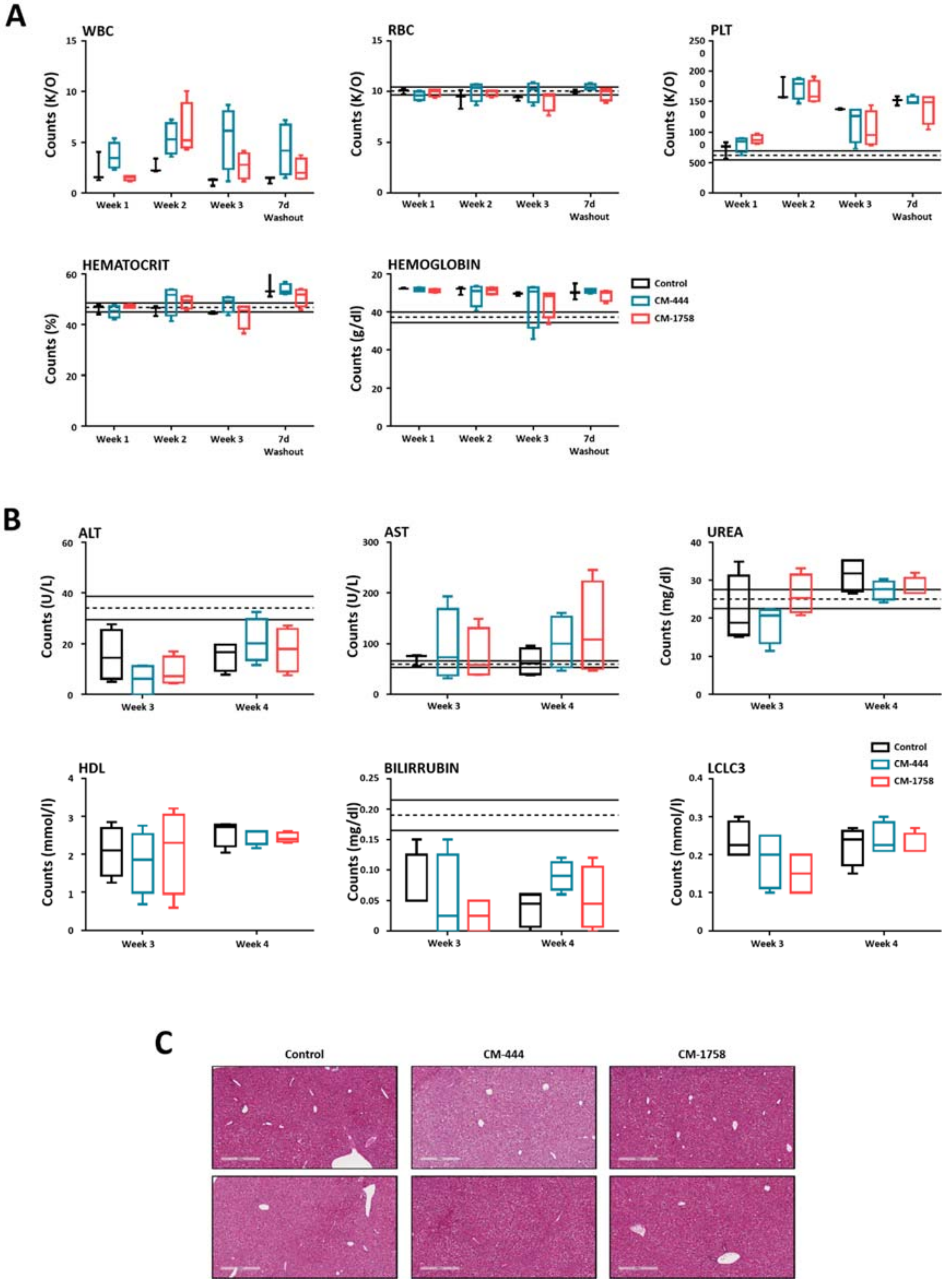

Supplementary Figure 3: CM-444 and CM-1758 do not show hematological or liver toxicity *in vivo*. **A)** Hematological parameters in healthy Rag2$^{-/-}$ γc$^{-/-}$ mice treated with vehicle (80% saline, 10% Tween 20, and 10% DMSO), 10 mg/kg i.p. of CM-444 and CM-1758 i.p. daily for 3 weeks (n = 4) followed by a 7-day washout period. WBC: white blood cells; RBC: red blood cells; PLT: platelet count. Box plots with centerline = median, box = 25 and 75% quantiles and whiskers = maximum and minimum values. **B)** Hepatic parameters in healthy Rag2$^{-/-}$γc$^{-/-}$ mice treated with vehicle (80% saline, 10% Tween 20, and 10% DMSO), 10 mg/kg i.p. of CM-444 and CM-1758 i.p. daily for 3 weeks (n =

4) followed by a 7-day washout period. ALB: albumin; ALP: alkaline phosphatase; AST: aspartate transaminase; ALT: alanine transaminase. Box plots with centerline = median, box = 25 and 75% quantiles and whiskers = maximum and minimum values. **C)** Hematoxylin and eosin staining for liver tissue in healthy Rag2$^{-/-}$ɣc$^{-/-}$ mice treated with vehicle (80% saline, 10% Tween 20, and 10% DMSO), 10 mg/kg i.p. of CM-444 and CM-1758 i.p. daily for 3 weeks (n = 4) followed by a 7-day washout period. Scale bar, 500µm. Source data are provided as a Source data file.

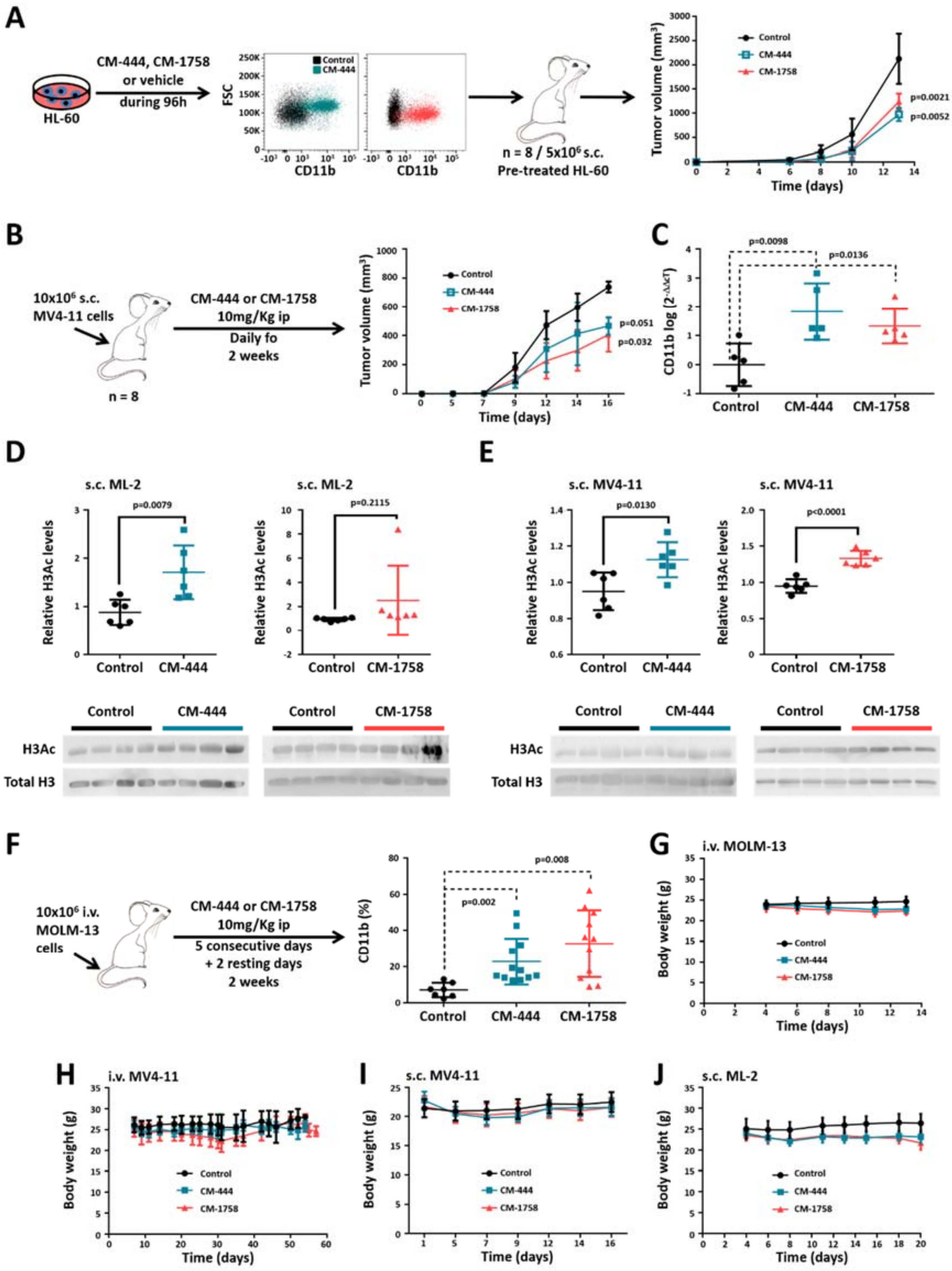

Supplementary Figure 4: CM-444 and CM-1758 induction of differentiation and anti-leukemic activity *in vivo.* A) HL-60 cells were pretreated *in vitro* with 270 nM of CM-444 or 300 nM of CM-1758 for 96 hours. After verifying CD11b induction by flow cytometry, equal amount of cells were injected subcutaneously in Rag2$^{-/-}$γc$^{-/-}$ mice, and tumor volumes were measured (n = 7). Error bars indicate the S.D. Statistical significance was calculated by a two-tailed Student's *t*-test. **B)** Schematic diagram of *in vivo* CM-444 and CM-1758 treatment procedure and tumor volume curve of MV4-11 subcutaneous xenograft model in Rag2$^{-/-}$γc$^{-/-}$ mice (n = 10). Error bars indicate the S.D.

Statistical significance was calculated by a two-tailed Student's *t*-test. **C)** *CD11b* from tumors of the s.c. MV4-11 model was measured by q-PCR. Error bars indicate the S.D. Statistical significance was calculated by a two-tailed Student's *t*-test. **D–E)** Relative H3Ac/H3 total levels after *in vivo* CM-444 and CM-1758 treatment in s.c. ML-2 (n=6) (**D**) and MV4-11 (n=6) (**E**) mouse model. Error bars indicate the S.D. Statistical significance was calculated by a two-tailed Student's *t*-test. The original western blot as shown below. **F)** Schematic diagram of *in vivo* CM-444 and CM-1758 treatment procedure and CD11b levels in blood samples of Rag2$^{-/-}$ɣc$^{-/-}$ mice engrafted i.v. with MOLM-13 cells (n = 11). Error bars indicate the S.D. Statistical significance was calculated by a two-tailed Student's *t*-test. **G–J)** Mean ± SEM body weight of mice of i.v. MOLM-13; (**G**) i.v. MV4-11; (**H**) s.c. MV4-11 (**I**) and s.c. ML-2; and (**J**) AML mouse models. Uncropped blots and source data are provided as a Source data file.

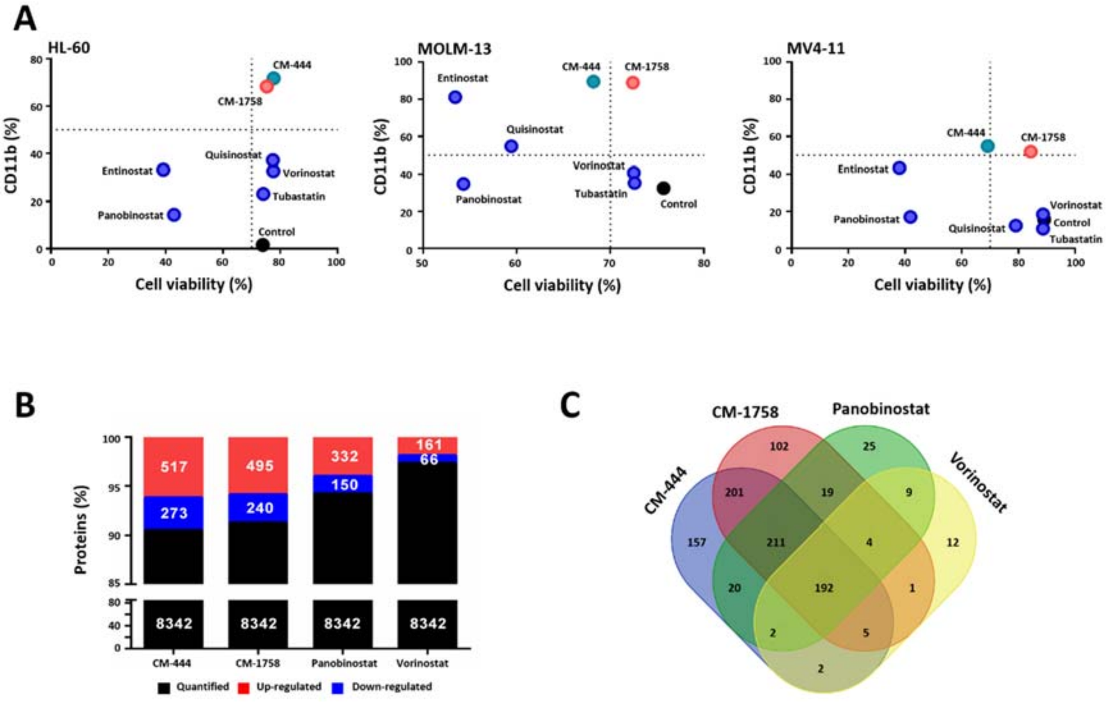

Supplementary Figure 5: Proteome analysis after CM-444 and CM-1758 treatment of AML cells. A) Cell differentiation assay measuring CD11b by flow cytometry in HL-60, MOLM-13, and MV4-11 cell lines treated with 25% GI_{50} of CM-444, CM-1758, and the commercial HDACi, Panobinostat, Vorinostat, Entinostat, Quisinostat, or Tubastatin for 48 h. The data shown are the mean of three biologically independent experiments. **B)** Total number of proteins quantified and the fraction proteins regulated by individual HDACi. The bar chart shows the percentage of upregulated proteins (log FC > 0.3, $p < 0.01$, shown in red) and downregulated proteins (log FC < 0.3, $p > 0.01$, shown in blue). **C)** Venn diagram of deregulated proteins in ML-2 cells after treatment with CM-444, CM-1758, Panobinostat, or Vorinostat compared with untreated cells. Source data are provided as a Source data file.

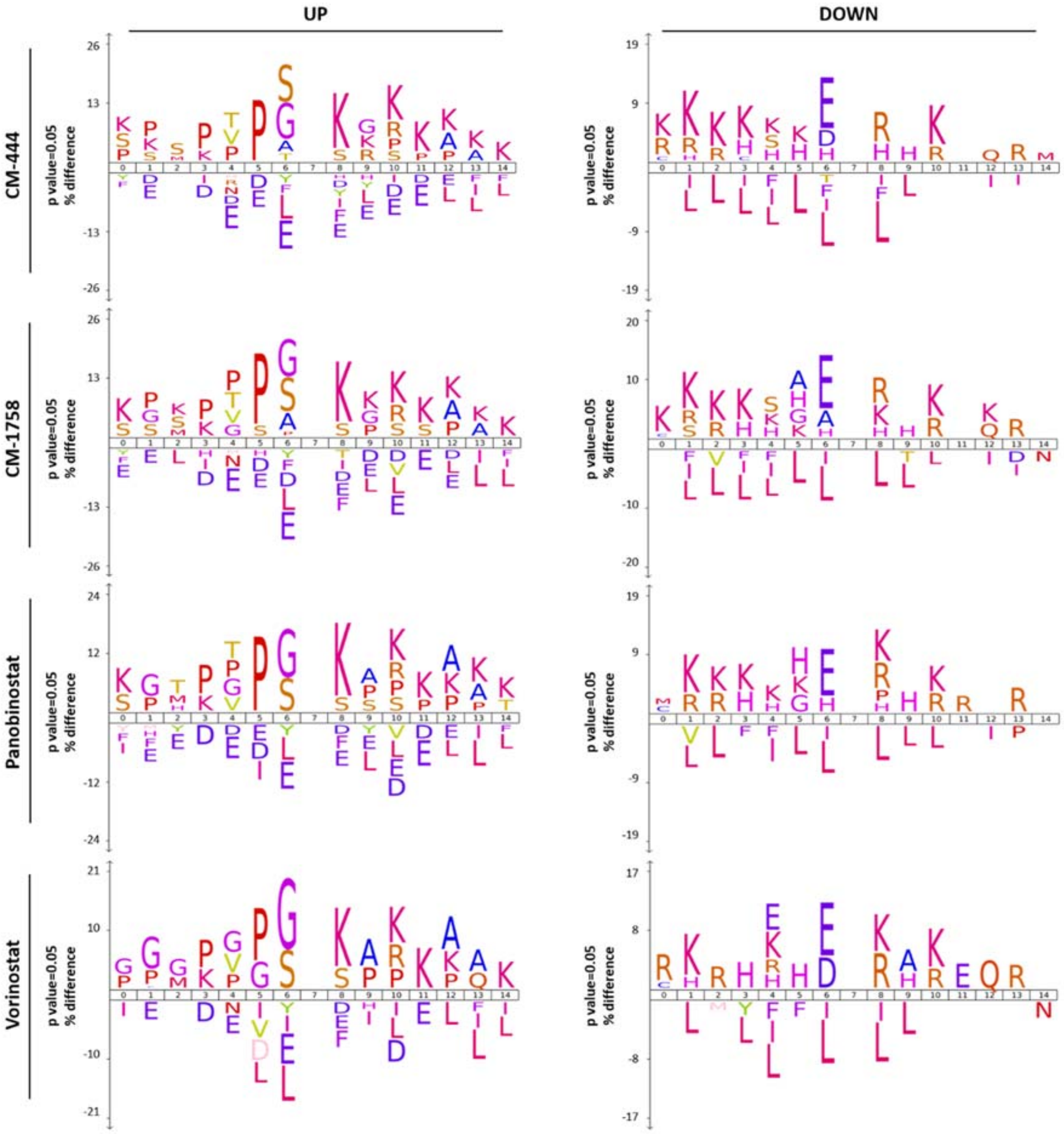

Supplementary Figure 6: Analysis of the acetylation motifs. Sequence motif surrounding acetylated lysines after treatment of AML cells with CM-444, CM-1758, Panobinostat, or Vorinostat. The logo was created using the icelogo software package. A cutoff value of $p < 0.01$ was used.

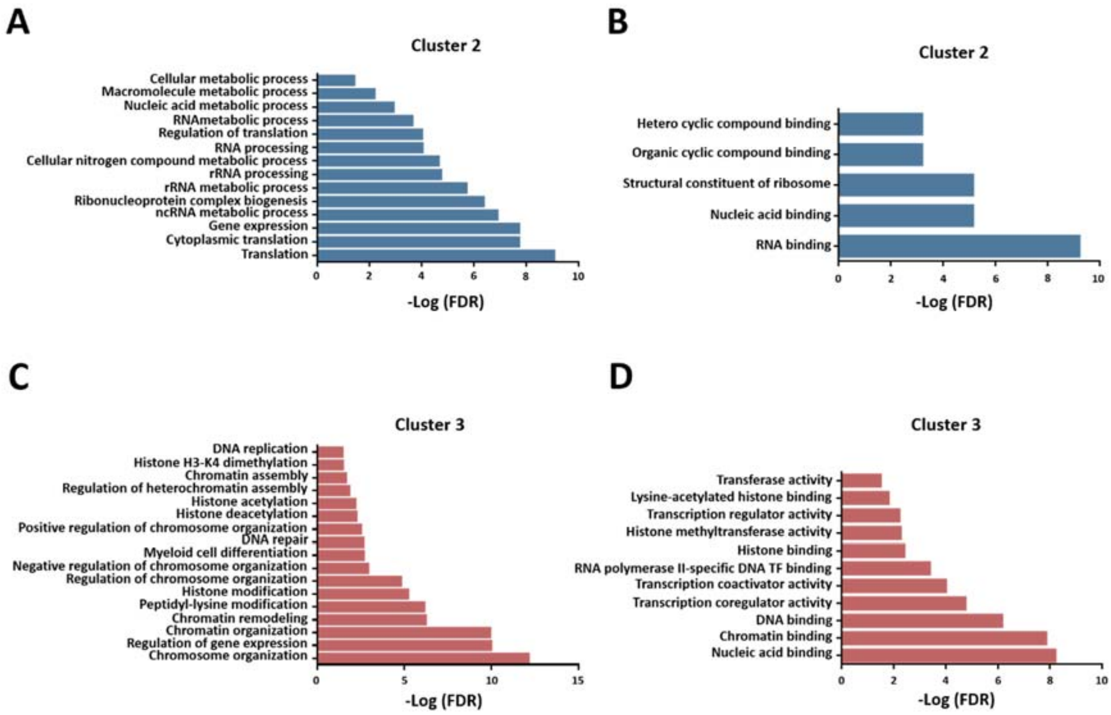

Supplementary Figure 7: Gene ontology (GO) analysis of the three functionally different clusters generated from the Acetyl-K sites specifically deregulated by CM-444 and CM-1758. A) Biological process GO results of cluster 2. **B)** Molecular function GO results of cluster 2. **C)** Biological process GO results of cluster 3. **D)** Molecular function GO results of cluster 3. Source data are provided as a Source data file.

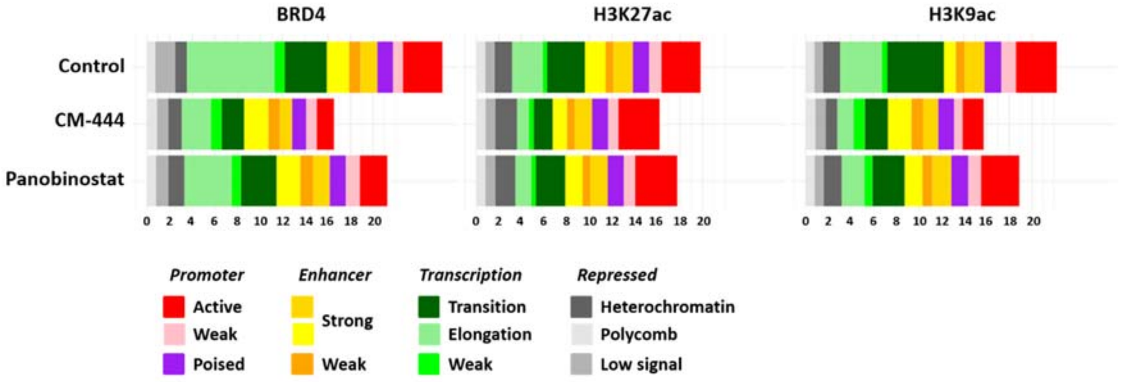

Supplementary Figure 8: Distribution of the different chromatin states in untreated cells and cells treated with CM-444 and Panobinostat for BRD4 and the histone marks H3K27ac and H3K9ac from Cut&Run peaks.

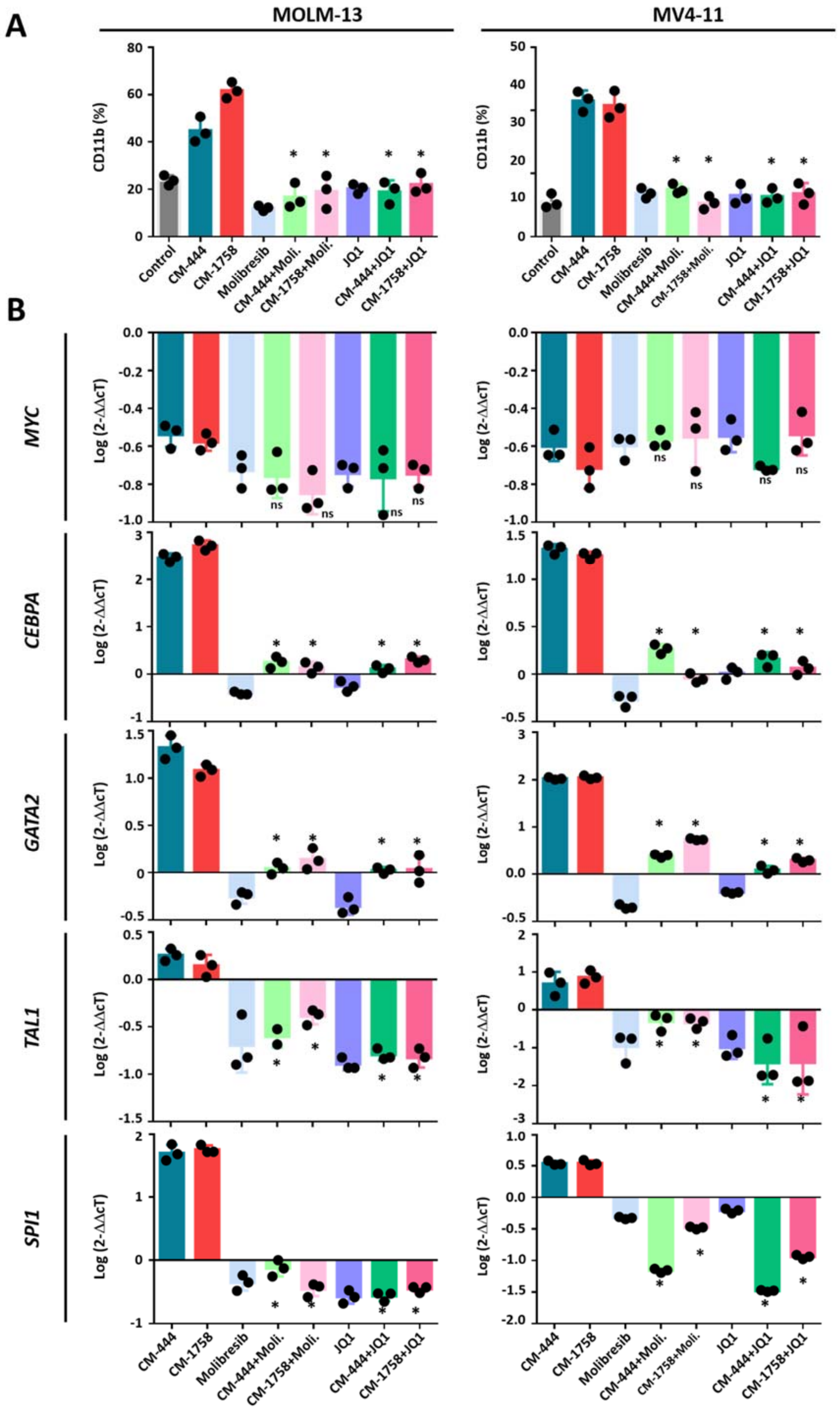
A
MOLM-13
MV4-11
CD11b (%)
Control
CM-444
CM-1758
Molibresib
CM-444+Moli.
CM-1758+Moli.
JQ1
CM-444+JQ1
CM-1758+JQ1
B
MYC
CEBPA
GATA2
TAL1
SPI1
Log (2-ΔΔcT)
ns

Supplementary Figure 9: Bromodomains have an important role in the differentiation therapy exerted by CM-444 or CM-1758 in AML cells. A) Cell differentiation assay measuring CD11b by flow cytometry after treating MOLM-13 (left) and MV4-11 (right) cells daily with 25% GI_{50} of CM-444, CM-1758, Molibresib, JQ1, and the combination of CM-444 or CM-1758 with Molibresib or JQ1 for 48 h. Error bars indicate the S.D. of three biological replicates. Statistical significance was calculated by a two-tailed Student's *t*-test. n.s. = non-significant; $^*p \leq 0.05$. **B)** q-PCR of *MYC*, *CEBPA*, *GATA2*, *TAL1* and *SPI1* after treating MOLM-13 (left) and MV4-11 (right) cells daily with 25% GI_{50} of CM-444, CM-1758, Molibresib, JQ1, and the combination of CM-444 or CM-1758 with Molibresib or JQ1 for 48 h. Data are presented as mean values +/- S.D. of three biological replicates. Statistical significance was calculated by a two-tailed Student's *t*-test. n.s. = non-significant; $^*p \leq 0.05$. Source data are provided as a Source data file.

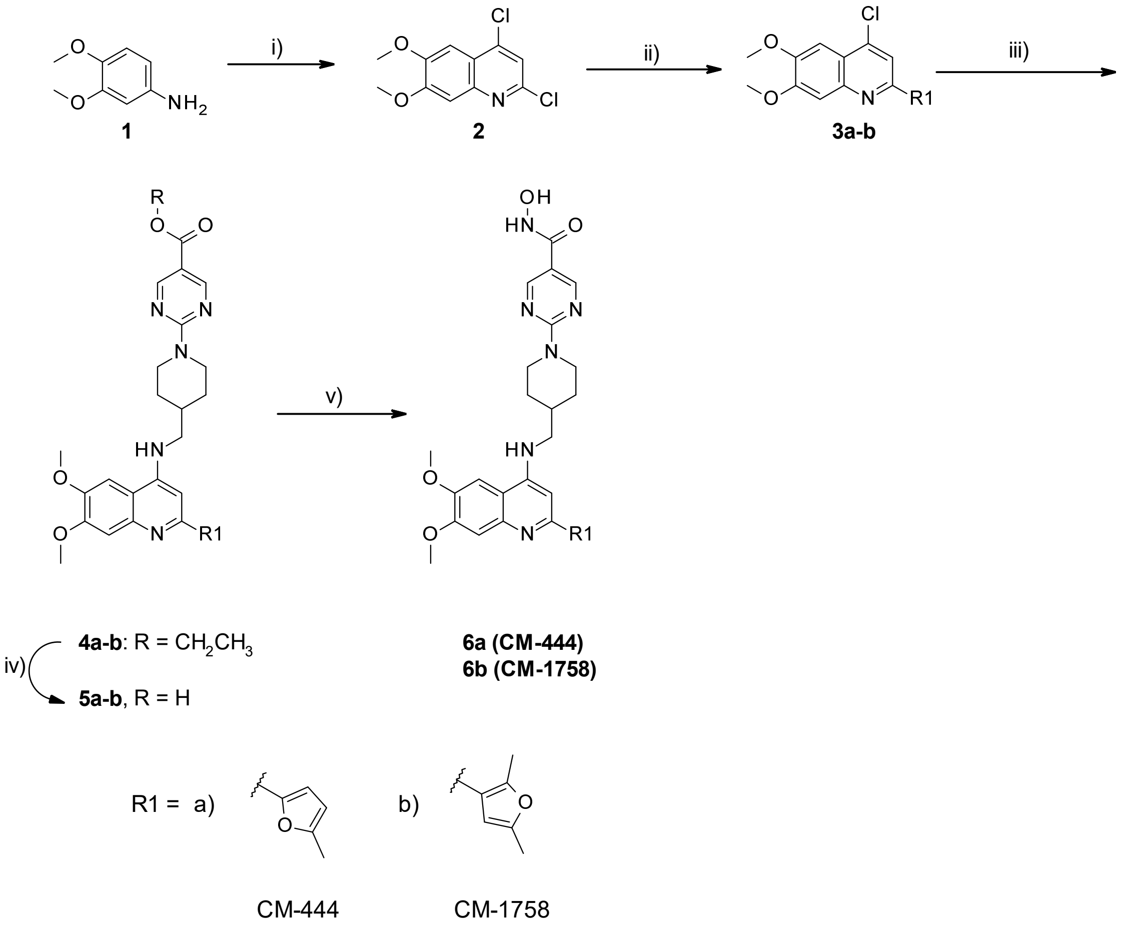

Supplementary Figure 10: Synthetic scheme for the synthesis of compounds CM-444 and CM-1758. 1: 3,4-dimethoxyaniline; **2**: 2,4-Dichloro-6,7-dimethoxy-quinoline; **3a**: 4-Chloro-6,7-dimethoxy-2-(5-methyl-2-furyl)quinoline; **3b**: 4-chloro-2-(2,5-dimethyl-3-furyl)-6,7-dimethoxy-quinoline; **4a**: Ethyl 2-[4-[[[6,7-dimethoxy-2-(5-methyl-2-furyl)-4-quinolyl]amino]methyl]-1-piperidyl]pyrimidine-5-carboxylate; **4b**: 2-[4-[[[2-(2,5-dimethyl-3-furyl)-6,7-dimethoxy-4-quinolyl] amino]methyl]-1-piperidyl]pyrimidine-5-carboxylate; **5a**: 2-[4-[[[6,7-dimethoxy-2-(5-methyl -2-furyl)-4-quinolyl]amino]methyl]-1-piperidyl]pyrimidine-5-carboxylic acid; **5b**: 2-[4-[[[2-(2,5-dimethyl-3-furyl)- 6,7-dimethoxy-4-quinolyl]amino]methyl]-1-piperidyl]pyrimidine-5-carboxylic acid; **6a (CM-444)**: 2-[4-[[[6,7-dimethoxy-2-(5-methyl-2-furyl)-4-quinolyl]amino]methyl]-1-piperidyl]pyrimidine-5-carbohydroxamic acid; **6b (CM-1758)**: 2-[4-[[[2-(2,5-dimethyl-3-furyl)-6,7-dimethoxy-4-quinolyl]amino]methyl] -1-piperidyl]pyrimidine-5-carbohydroxamic acid. **i)** $POCl_3$, malonic acid, 100ºC, 16 h; **ii)** corresponding boronic ester, $Pd(PPh3)_4$, K_2CO_3, 1,4-dioxane/H2O (8:1 or 10:1), 90–100ºC, 12–16 h; **iii)** ethyl 2-[4-(aminomethyl)-1-piperidyl]pyrimidine-5-carboxylate, $Pd_2(dba)_3$, BINAP, Cs_2CO3, 1,4-dioxane, 120ºC, 12 h; **iv)** $LiOH{\cdot}H_2O$, THF/H_2O (2:1), 25ºC, 12 h; **v)** $THPONH_2$, HOBt, DIEA, EDCI, DMF, 25ºC, 12 h; then aqueous HCl (0.5 M or 2.0 M), room temperature.

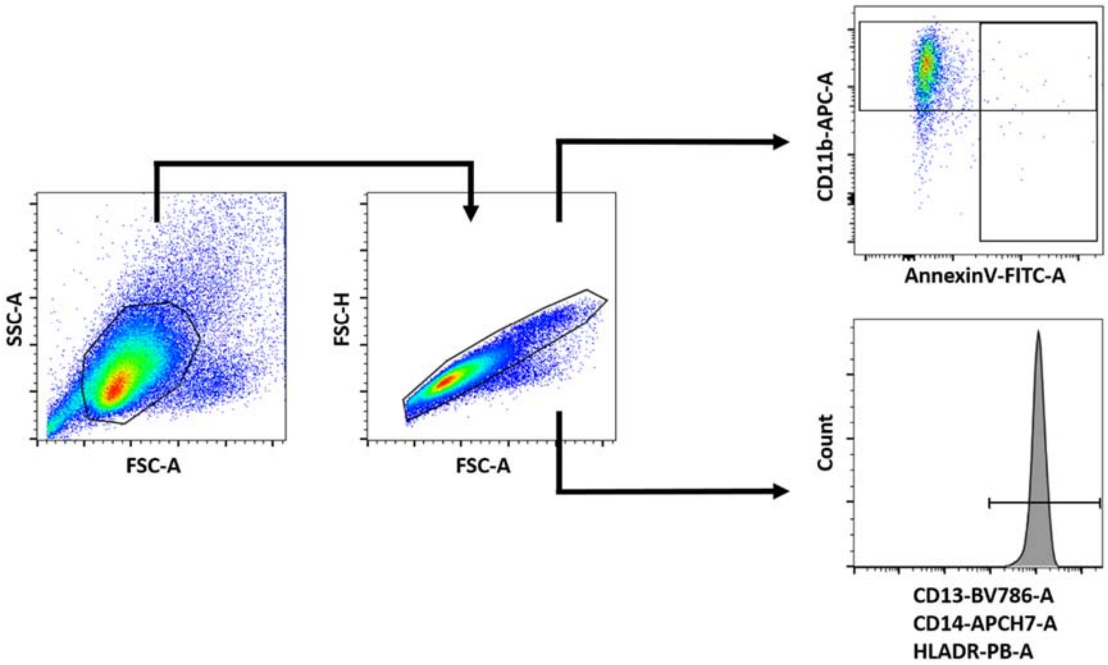

Supplementary Figure 11: Gating strategy for flow cytometry of CD11b / Annexin-v analysis (upper panel) and of the rest of myeloid markers (lower panel) in AML cells.

Supplementary Table 1: analysis of surface myeloid markers after CM-444 and CM-1758 treatment.

Cell line	Treatment	CD11b (%)	CD13 (%)	CD14 (%)	HLA-DR (%)
HL-60	Control	11.7	99.6	2.35	2.48
	CM-444	74.3	100	21.5	9.16
	CM-1758	71.2	100	18.9	12.2
ML-2	Control	15	77.3	2.10	4.38
	CM-444	80.1	100	5.20	35.7
	CM-1758	75.6	98.6	2.86	17.4
MOLM-13	Control	39.6	10.5	18.5	10.4
	CM-444	92.8	49.3	66.5	35.1
	CM-1758	93.0	54.8	35.6	27.9
MV4-11	Control	10.2	62.7	12.3	12.5
	CM-444	58.9	98.0	57.6	42.1
	CM-1758	57.9	98.1	56.2	38.4

Supplementary Table 2: cell differentiation of AML patient samples with CM-444 and CM-1758 treatment.

Sample	Genetic alterations	CD11b-positive cells (%)					
		Control	CM-444 (nM)		CM-1758 (nM)		ATRA (µM)
			200	500	200	500	2
AML-1	46,XY,del(5)(q13q31),i(17)(q10)	23.3	35.2	46.6	30.6	43.2	26.1
AML-2	45,X,-X,t(8;21)(q22;q22) Mut: cKIT	13.8	16.8	31.7	15.2	30.1	17.5
AML-3	inv(16)(p13;q22) CBFb-MYH11	9.0	12.3	20.5	15.5	23.7	10.3
AML-4	Mut: FLT3-ITD, NPM1, DNMT3A	15.9	20.8	39.5	21.9	38.4	25.4
AML-5	Mut: NPM1, FLT3-ITD, TET2, DNMT3A	10.6	20.2	32.1	19.2	30.1	15.2
AML-6	Mut: FLT3-V579A, RUNX1, SRP72, DMT3A	12.9	29.7	38.6	27.2	35.2	16.3
AML-7	Mut: NPM1, DNMT3A, PTPN11	25.4	32.9	49.2	30.1	35.5	29.3
AML-8	Mut: SRSF2, IDH1, CALR	10.2	15.3	23.7	16.2	25.5	13.3

Supplementary Table 3: ADME and Cardiovascular safety profiles of CM-444 and CM-1758.

ADME		
	**CM-444**	**CM-1758**
$P450_S$ Inhibition	58.8% (1A2)<br>0% (2C9)<br>5.6% (2C19)<br>10.8% (2D6)<br>50.8% (3A4)	55.4% (1A2)<br>12.2% (2C9)<br>23.7% (2C19)<br>13.1% (2D6)<br>46.9% (3A4)
Plasma protein binding<br>% Unbound	1.1 ± 0.1 (Human)<br>N.C. (Mouse)	1.0 ± 0.0 (Human<br>N.C. (Mouse)
Solubility (at pH = 7.4)	117.6 µg/ml	> 144.1 µg/ml
PAMPA (Pe, 10^{-6} in nm/s)	5.04	6.74
Liver Microsomal Stability<br>Estimation $T_{1/2}$ (min)	28.8 (Human)<br>18.4 (Mouse)	37.9 (Human)<br>12.5 (Mouse)
Liver Hepatocytes<br>Estimation $T_{1/2}$ (min)	71.1 (Human)<br>22.8 (Mouse)	92.1 (Human)<br>19.5 (Mouse)
CACO-2 Permeability (A to B direction and efflux)	< 0.01 (P_{app} 10^{-6} cm/s A to B)<br>Efflux ratio > 1046.22	< 0.01 (P_{app} 10^{-6} cm/s A to B)<br>Efflux ratio > 1046.22
**Cardiovascular Safety**		
	**CM-444**	**CM-1758**
hERG binding: IC_{50} (µM)	16.84 (hill slope of 1.33)	4.89 (hill slope of 0.99)

N.C. = not calculated due to poor recovery.

Supplementary Table 4: LC_{50} values on cytotoxicity assay.

Cell type	CM-444 LC_{50} (μM)	CM-1758 LC_{50} (μM)
THLE-2	0.794	0.779
PBMCs	1.240	2.410

Supplementary Table 5: CM-444 plasmatic concentrations after intraperitoneal (i.p.) administration (10 mg/kg) (n= 4).

Time (h)	[CM-444] (nM)		
	**Mean**	**SD**	**RSD (%)**
0.25	915.7	192.4	21.0
1	202.6	72.1	35.6
2	27.7	11.0	39.6
4	5.0	0.7	14.4
8	2.4	0.8	33.1
24	0.9	0.4	45.4

Supplementary Table 6: CM-1758 plasmatic concentrations after intraperitoneal (i.p.) administration (10 mg/kg) (n= 4). BLOQ: below limit of quantification.

Time (h)	[CM-1758] (nM)		
	**Mean**	**SD**	**RSD (%)**
0.25	1695.5	406.6	24.0
1	352.3	169.0	48.0
2	77.5	50.9	65.7
4	17.1	2.8	16.7
8	4.3	0.8	19.4
24	BLOQ		

Supplementary Table 7: CM-444 pharmacokinetic parameters estimated by fitting previously reported experimental data (Supplementary Table 7) to a non-compartmental model (NCA) using Winnonlin software for pharmacokinetic analysis. The parameters are: Area Under the Curve computed to the last observation (AUClast); half-life of the product (T½); clearance (Cl); volume of distribution (Vss).

Parameter	Mean	SD	%RSD
**AUClast (nM*h)**	916.15	164.7	18.0
**t½(h)***	8.46	1.7	20.6
**Cl/F (L/h)****	0.53	0.1	17.7
**Vz/F (L)****	6.56	2.0	30.2
**Norm. Vz/F (L/kg)**	262.3		
**Norm. Cl/F (L/h·kg)**	21.3		

Supplementary Table 8: CM-1758 pharmacokinetic parameters estimated by fitting previously reported experimental data (Supplementary Table 8) to a non-compartmental model (NCA) using Winnonlin software for pharmacokinetic analysis. The parameters are: Area Under the Curve computed to the last observation (AUClast); half-life of the product (T½); clearance (Cl); volume of distribution (Vss).

Parameter	Mean	SD	%RSD
**AUClast (nM*h)**	1694.25	478.3	28.2
**t½(h)***	1.95	0.2	10.9
**Cl/F (L/h)****	0.26	0.1	31.5
**Vz/F (L)****	0.72	0.2	21.0
**Norm. Vz/F (L/kg)**	28.6		
**Norm. Cl/F (L/h·kg)**	10.4		

Supplementary Table 9: GI_{50} data of AML cell lines for HDAC and BRDs inhibitors.

Cell line	Panobinostat (nM)	Vorinostat (μM)	Entinostat (μM)	Quisinostat (nM)	Tubastatin (μM)	JQ1 (μM)	Molibresib (μM)
**HL-60**	18	5.2	7.8	112	> 10	> 10	> 10
**ML-2**	51.6	4.5	9.1	76	> 10	> 10	> 10
**MV4-11**	13	2.5	3	13	> 10	> 10	> 10
**MOLM-13**	32	3.2	2.4	67	> 10	> 10	> 10

Supplementary Table 10: Primers used for Q-PCR.

Gene	Primer sequence
MYC	F: TGCTCCATGAGGAGACACC
	R: CTCTGACCTTTTGCCAGGAG
CDKN2A	F: GTCGCAGGTTCTTGGTCACT
	R: CGAATCTGCACCGTAGTTGA
CDKN1A	F: CCATGTGGACCTGTCACTGT
	R: CGGCGTTTGGAGTGGTAGAA
GATA2	F: GCCATAAGGTGGTGGTTGTC
	R: CTACCTCTGCAATGCCTGTG
TAL1	F: AGGAGACCTTCCCCCTATGA
	R: CCCCGTTCACATTCTGCT
CEBPA	F: GGATAACCTTGTGCCTTGGA
	R: GGCAGGAAACCTCCAAATAA
SPI1	F: TGCCCTATGACACGGATCTA
	R: GGGGTGGAAGTCCCAGTAAT
ITGAM	F: GCTCTGCTTCCTGTTTGGAT
	R: TGCTACCAGAGCCATCAATC
GUS	F: GAAAATATGTGGTTGGAGAGCTCATT
	R: CCGAGTGAAGATCCCCTTTTTA

Supplementary Table 11: Calculation of the interval confidence using the Wichura algorithm.

p-value	< Z-score
...	...
0.20	[-1.28 σ; 1.28 σ]
0.10	[-1.65 σ; 1.65 σ]
0.05	[-1.96 σ; 1.96 σ]
0.02	[-2.33 σ; 2.33 σ]
0.01	[-2.58 σ; 2.58 σ]
0.005	[-2.81 σ; 2.81 σ]
...	...
