# Supplementary material for: Epigenetic-based differentiation therapy for Acute Myeloid Leukemia
Source: Nat Commun. 2024 Jul 2;15:5570. doi: 10.1038/s41467-024-49784-y (PMC11219871; doi:10.1038/s41467-024-49784-y)
Supplement: Supplementary file 3 — Description of Additional Supplementary Files [file 41467_2024_49784_MOESM3_ESM.pdf]

Description of Additional Supplementary Files

File Name: Supplementary Data 1
Description: Information about the epigenetic small molecules used in the initial dual differentiation-apoptosis assay screening in HL-60 cell line.

File Name: Supplementary Data 2
Description: **Percentage of inhibition of compounds CM-444 and CM-1758 at 10µm against a panel of 95 epigenetic targets.** IC_{50} values are indicated when the percentage of inhibition is higher than 50%. HDM= Histone Demethylase; HAT= Histone Acetyltransferase; HMT= Histone Methyltransferase; BRD= Bromodomains; DNMT= DNA Methyltransferase; HDAC= Histone Deacetylase

File Name: Supplementary Data 3
Description: **Mutations and GI50 data of AML cell lines.**
