# Supplementary material for: Epigenetic-based differentiation therapy for Acute Myeloid Leukemia
Source: Nat Commun. 2024 Jul 2;15:5570. doi: 10.1038/s41467-024-49784-y (PMC11219871; doi:10.1038/s41467-024-49784-y)
Supplement: Supplementary file 5 — Reporting Summary [file 41467_2024_49784_MOESM5_ESM.pdf]

nature portfolio

Corresponding author(s): Julen Oyarzabal , Xabier Agirre and Felipe Prósper

Last updated by author(s): Jun 18, 2024

Reporting Summary

Nature Portfolio wishes to improve the reproducibility of the work that we publish. This form provides structure for consistency and transparency in reporting. For further information on Nature Portfolio policies, see our Editorial Policies and the Editorial Policy Checklist.

Statistics

For all statistical analyses, confirm that the following items are present in the figure legend, table legend, main text, or Methods section.

n/a	Confirmed	
☐	☒	The exact sample size (*n*) for each experimental group/condition, given as a discrete number and unit of measurement
☐	☒	A statement on whether measurements were taken from distinct samples or whether the same sample was measured repeatedly
☐	☒	The statistical test(s) used AND whether they are one- or two-sided<br>*Only common tests should be described solely by name; describe more complex techniques in the Methods section.*
☐	☒	A description of all covariates tested
☐	☒	A description of any assumptions or corrections, such as tests of normality and adjustment for multiple comparisons
☐	☒	A full description of the statistical parameters including central tendency (e.g. means) or other basic estimates (e.g. regression coefficient) AND variation (e.g. standard deviation) or associated estimates of uncertainty (e.g. confidence intervals)
☐	☒	For null hypothesis testing, the test statistic (e.g. *F*, *t*, *r*) with confidence intervals, effect sizes, degrees of freedom and *P* value noted<br>*Give P values as exact values whenever suitable.*
☒	☐	For Bayesian analysis, information on the choice of priors and Markov chain Monte Carlo settings
☒	☐	For hierarchical and complex designs, identification of the appropriate level for tests and full reporting of outcomes
☒	☐	Estimates of effect sizes (e.g. Cohen's *d*, Pearson's *r*), indicating how they were calculated

Our web collection on statistics for biologists contains articles on many of the points above.

Software and code

Policy information about availability of computer code

Data collection	RNA-seq and CUT&RUN data generated in this study have been deposited in NCBI Gene Expression Omnibus (GEO) repository under the accession numbers GSE219230 (https://www.ncbi.nlm.nih.gov/geo/query/acc.cgi?acc=GSE219230) and GSE268008 (https://www.ncbi.nlm.nih.gov/geo/query/acc.cgi?acc=GSE268008), respectively.<br>The mass spectrometry proteomics data generated in this study have been deposited in the ProteomeXchange Consortium via the PRIDE partner repository with the data set identifier PXD050623 (http://www.ebi.ac.uk/pride/archive/projects/PXD050623).
Data analysis	The commercial software used for data analysis is properly described in the "Materials and Methods" section. FlowJo software; GoldSuite 5.3.6 program (Cambridge Crystallographic Data Centre, https://www.ccdc.cam.ac.uk/pages/Home.aspx); bcl2fastq2 Conversion Software v2.19 (Illumina); FastQC (Bioinformatics Babraham Institute); Bowtie 2 (Johns Hopkins University); quant3p script (github.com/ctlab/quant3p); DEseq2 package (R); Database for Annotation, Visualization, and Integrated Discovery database (david.ncifcrf.gov/home.jsp); Gene Set Enrichment Analyses 3.0 (Broad Institute and UC San Diego);GraphPad prism 6.0; PyroQ-CpG analysis software 1.0.9; ImageLab 6.0.1 BioRad; MaxQuant 1.6.1.0; Prostar (v1.18.5) (Wieczorek et al., 2017); STRING database (https://string-db.org/, version 11.5).

For manuscripts utilizing custom algorithms or software that are central to the research but not yet described in published literature, software must be made available to editors and reviewers. We strongly encourage code deposition in a community repository (e.g. GitHub). See the Nature Portfolio guidelines for submitting code & software for further information.

Data

Policy information about availability of data

All manuscripts must include a data availability statement. This statement should provide the following information, where applicable:

- Accession codes, unique identifiers, or web links for publicly available datasets
- A description of any restrictions on data availability
- For clinical datasets or third party data, please ensure that the statement adheres to our policy

RNA-seq and CUT&RUN data generated in this study have been deposited in NCBI Gene Expression Omnibus (GEO) repository under the accession numbers GSE219230 (https://www.ncbi.nlm.nih.gov/geo/query/acc.cgi?acc=GSE219230) and GSE268008 (https://www.ncbi.nlm.nih.gov/geo/query/acc.cgi?acc=GSE268008), respectively.
The mass spectrometry proteomics data generated in this study have been deposited in the ProteomeXchange Consortium via the PRIDE partner repository with the data set identifier PXD050623 (http://www.ebi.ac.uk/pride/archive/projects/PXD050623).
The remaining data are available within the article, Supplementary Information or Source data file. Source data are provided with this paper.

Human research participants

Policy information about studies involving human research participants and Sex and Gender in Research.

Reporting on sex and gender	We do not repport this type of information.
Population characteristics	We have analyzed 10 samples derived from patients with acute myeloid leukemia. The characteristics of these samples are defined in Table S2 (Supplementary information) of the manuscript.
Recruitment	We have analyzed 10 samples derived from patients with acute myeloid leukemia with different genetic alterations.
Ethics oversight	This study was approved by the Clinical Research Ethics Committee of Clínica Universidad de Navarra.

Note that full information on the approval of the study protocol must also be provided in the manuscript.

Field-specific reporting

Please select the one below that is the best fit for your research. If you are not sure, read the appropriate sections before making your selection.

[x] Life sciences   [ ] Behavioural & social sciences   [ ] Ecological, evolutionary & environmental sciences

For a reference copy of the document with all sections, see nature.com/documents/nr-reporting-summary-flat.pdf

Life sciences study design

All studies must disclose on these points even when the disclosure is negative.

Sample size	No sample size was calculated. All availble samples were included in each analysis
Data exclusions	No data were excluded.
Replication	All experiments and measures were made at least in triplicate (specific replicates are given for each particular experiment) obtaining in all of the cases the similar results.
Randomization	All experiments made in mice assigned the different groups randomly (i.e.: treated or untreated).
Blinding	Investigator were blinded to group allocation during diverse analyses using mouse samples.

Reporting for specific materials, systems and methods

We require information from authors about some types of materials, experimental systems and methods used in many studies. Here, indicate whether each material, system or method listed is relevant to your study. If you are not sure if a list item applies to your research, read the appropriate section before selecting a response.

Materials & experimental systems

n/a	Involved in the study
☐	☒ Antibodies
☐	☒ Eukaryotic cell lines
☒	☐ Palaeontology and archaeology
☐	☒ Animals and other organisms
☒	☐ Clinical data
☐	☒ Dual use research of concern

Methods

n/a	Involved in the study
☒	☐ ChIP-seq
☐	☒ Flow cytometry
☒	☐ MRI-based neuroimaging

Antibodies

Antibodies used	All antibodies were from commercial sources and are properly descibed in "Materials and Methods" section.<br>For Western Blot: Acetyl H3 (rabbit polyclonal antibody, Cat No 06-599, Millipore) diluted 1:50,000 , H3K27 me3 (mouse monoclonal antibody to histone H3 trimethyl K27, Cat No ab6002, Abcam) diluted 1:2000. Total H3 was used as a loading control (diluted 1:50,000) (Anti-Histone H3, CT, pan, rabbit polyclonal, Cat No 07-690, Millipore).<br>For Dot Blot, Monoclonal antibody 5-Methylcytidine, Cat No BI-MECY-1000, Eurogentec, diluted 1:4000 .<br>For flow cytometry: 8 µl per sample of APC-CD11b (BD Pharmingen, Franklin Lakes, NJ, USA), 1 µl per sample of FITC annexin-V (AV) (BD Pharmingen, Franklin Lakes, NJ, USA), 2 µl per sample of BV786-CD13 (Cat No 744748, BD Pharmingen, Franklin Lakes, NJ, USA), 5 µl per sample of APCH7-CD14 (Cat No 641394, BD Pharmingen, Franklin Lakes, NJ, USA) and 1 µL of diluted 1:10 antibody PB-HLADR (Cat No 307633, Biolegend).<br>For CUT&RUN: Acetyl-Histone H3 (Lys 9) (C5B11) diluted 1:50, rabbit monoclonal, Cat No 9649T, Cell Signaling; Acetyl-Histone H3 (Lys 27) (D5E4) XP diluted 1:100, rabbit monoclonal, Cat No 8173T, Cell Signaling; 0.5µg of BRD4 CUTANA CUT&RUN antibody, Cat No 13-2003, Epicypher.
Validation	The antibodies employed in our study were validated by the manufacturers and used according to the manufacturers' instructions.

Eukaryotic cell lines

Policy information about cell lines and Sex and Gender in Research

Cell line source(s)	The cell lines were obtained from the DSMZ or the American Type Culture Collection (ATCC):<br>KASUMI-1 (ACC 220, DSMZ), HL-60 (ACC 3, DSMZ), NB-4 (ACC 207, DSMZ), OCI-AML3 (ACC 582, DSMZ), MV4-11 (ACC 102, DSMZ), MOLM-13 (ACC 554, DSMZ), HEL (ACC 11, DSMZ), GF-D8 (ACC 615, DSMZ), TF-1 (CRL-2003, ATCC), THP-1 (TIB-202, ATCC), M-O7e (ACC 104, DSMZ), OCI-AML2 (ACC 99, DSMZ), MONO-MAC-6 (ACC 124, DSMZ) and UT-7 (ACC 137, DSMZ).
Authentication	All cell lines were authenticated by performing an short tandem repeat allele profile, cells were grown for no more than 15 passages upon thawing.
Mycoplasma contamination	All cell lines were tested for mycoplasma (MycoAlert Sample Kit, Cambrex).
Commonly misidentified lines<br>(See ICLAC register)	None of the cell lines used is reported at ICLAC register.

Animals and other research organisms

Policy information about studies involving animals; ARRIVE guidelines recommended for reporting animal research, and Sex and Gender in Research

Laboratory animals	For in vivo experiments female BALB/cA Rag2−/−γc−/− mice between 6 and 8 weeks of age were used.
Wild animals	The study does not involve wild animals.
Reporting on sex	Due to engraftment of the leukemia cells, we have only used female mice.
Field-collected samples	The study does not involve samples collected from the field.
Ethics oversight	All animal studies had previous approval from the Animal Care and Ethics Committee of the University of Navarra.

Note that full information on the approval of the study protocol must also be provided in the manuscript.

Dual use research of concern

Policy information about dual use research of concern

Hazards

Could the accidental, deliberate or reckless misuse of agents or technologies generated in the work, or the application of information presented in the manuscript, pose a threat to:

No	Yes	
☒	☐	Public health
☒	☐	National security
☒	☐	Crops and/or livestock
☒	☐	Ecosystems
☒	☐	Any other significant area

Experiments of concern

Does the work involve any of these experiments of concern:

No	Yes	
☒	☐	Demonstrate how to render a vaccine ineffective
☒	☐	Confer resistance to therapeutically useful antibiotics or antiviral agents
☒	☐	Enhance the virulence of a pathogen or render a nonpathogen virulent
☒	☐	Increase transmissibility of a pathogen
☒	☐	Alter the host range of a pathogen
☒	☐	Enable evasion of diagnostic/detection modalities
☒	☐	Enable the weaponization of a biological agent or toxin
☒	☐	Any other potentially harmful combination of experiments and agents

Flow Cytometry

Plots

Confirm that:

☒ The axis labels state the marker and fluorochrome used (e.g. CD4-FITC).

☒ The axis scales are clearly visible. Include numbers along axes only for bottom left plot of group (a 'group' is an analysis of identical markers).

☐ All plots are contour plots with outliers or pseudocolor plots.

☒ A numerical value for number of cells or percentage (with statistics) is provided.

Methodology

Sample preparation

For CD11b/annexin-V detection by flow cytometry: 100,000 cells of ML-2, KASUMI-1, HL-60, NB-4, OCI-AML3, MV4-11, MOLM-13, HEL, GF-D8, TF-1, THP-1, M-O7e, OCI-AML2, MONO-MAC-6, and UT-7 cell lines were cultured at a density of 1 ×106 cells/mL and treated daily for 48 h with CM-444, CM-1758, or ATRA at (see Supplementary Table 2 for GI50 data). The cell lines HL-60, ML-2, MV4-11, and MOLM-13 were also treated daily up to 8 days with CM-444 and CM-1758 at 25% GI50. HL-60, ML-2, MOLM-13, and MV4-11 cell lines were also treated for 48 h with 25% GI50 of panobinostat, quisinostat, entinostat, vorinostat, or tubastatin (Table S11) or with the BRD inhibitors JQ1, molibresib, and the combination of CM-444 or CM-1758 with JQ1 and molibresib (Table S11). Next, the cells were washed twice with phosphate-buffered saline (PBS) and resuspended in 1X Binding Buffer at a concentration of 1 × 106 cells/mL. An 8-µL aliquot of APC-CD11b (BD Pharmingen, Franklin Lakes, NJ, USA) and 1 µL of FITC annexin-V (AV) (BD Pharmingen, Franklin Lakes, NJ, USA) antibodies were added and incubated for 15 min at room temperature in the dark. Finally, after addition of 400 µL of 1X Binding Buffer to each tube, samples were collected on a BD FACSCanto flow cytometer (Becton Dickinson, San Jose, CA, USA) and analyzed using FlowJo software.
For cell-cycle analysis, 250,000 cells of HL-60, ML-2, MV4-11, and MOLM-13 cell lines were cultured at a density of 1 × 106 cells/mL and treated for 24 hours with CM-444 and CM-1758. Next, the cells were washed twice with PBS and resuspended in 0.2% Tween 20 in PBS and 0.5 mg/mL Rnase A (Ribonuclease A Type III-A from bovine pancreas, Cat No. R5125, Sigma), and then incubated for 30 min at 37°C. Subsequently, the cells were stained with 25 µg/mL of propidium iodide (Cat No P4170, Sigma).

Instrument

BD FACSCanto flow cytometer (Becton Dickinson, San Jose, CA, USA)

Software	FLOWJO
Cell population abundance	Not applicable
Gating strategy	The detailed gating strategy of flow cytometry is shown in Supplementary Figure 11.

☒ Tick this box to confirm that a figure exemplifying the gating strategy is provided in the Supplementary Information.
